# Advances in Genes-Encoding Transporters for Cadmium Uptake, Translocation, and Accumulation in Plants

**DOI:** 10.3390/toxics10080411

**Published:** 2022-07-22

**Authors:** Jingyu Tao, Lingli Lu

**Affiliations:** 1 MOE Key Laboratory of Environment Remediation and Ecological Health, College of Environmental and Resource Sciences, Zhejiang University, Hangzhou 310058, China; taojingyu@zju.edu.cn; 2 Key Laboratory of Agricultural Resource and Environment of Zhejiang Province, College of Environmental and Resource Sciences, Zhejiang University, Hangzhou 310058, China

**Keywords:** Cadmium, transporters, Nramp, HMA, ABC, ZIP, YSL

## Abstract

Cadmium (Cd) is a heavy metal that is highly toxic for plants, animals, and human beings. A better understanding of the mechanisms involved in Cd accumulation in plants is beneficial for developing strategies for either the remediation of Cd-polluted soils using hyperaccumulator plants or preventing excess Cd accumulation in the edible parts of crops and vegetables. As a ubiquitous heavy metal, the transport of Cd in plant cells is suggested to be mediated by transporters for essential elements such as Ca, Zn, K, and Mn. Identification of the genes encoding Cd transporters is important for understanding the mechanisms underlying Cd uptake, translocation, and accumulation in either crop or hyperaccumulator plants. Recent studies have shown that the transporters that mediate the uptake, transport, and accumulation of Cd in plants mainly include members of the natural resistance-associated macrophage protein (Nramp), heavy metal-transporting ATPase (HMA), zinc and iron regulated transporter protein (ZIP), ATP-binding cassette (ABC), and yellow stripe-like (YSL) families. Here, we review the latest advances in the research of these Cd transporters and lay the foundation for a systematic understanding underlying the molecular mechanisms of Cd uptake, transport, and accumulation in plants.

## 1. Introduction

Cadmium (Cd) is a heavy metal that is highly toxic to animals and plants, ranking first among inorganic pollutants. Cd enters the soil–plant environment through natural processes and anthropogenic activities [1]. Natural processes include volcanic eruptions and soil erosion, and anthropogenic activities include power stations, heating systems, and urban transportation [2,3]. Soil pollution by heavy metals, including Cd, is essentially an irreversible process that may take hundreds of years to recover from. Cd accumulation in plants inhibits Fe(III) reductase activity, leading to Fe(II) deficiency that in turn affects photosynthesis [4]. Plants affected by Cd toxicity in polluted soils usually present retarded growth, chlorotic leaves, and brown root tips. Compared with other heavy metals, such as Pb, Cd is more soluble and easily absorbed by plants, and is subsequently accumulated in their edible parts, thus entering the food chain and posing a threat to humans [1]. An excessive intake of Cd in humans can damage the kidneys, leading to rhinitis, emphysema, and osteomalacia [5]. In recent years, Cd has become one of the major soil pollutants worldwide due to uncontrolled industrialization, unsustainable urbanization, and intensive agricultural practices. The itai—itai disease is the most serious chronic Cd poisoning caused by long-term oral consumption of Cd in Japan [6]. In China, Cd is the most severe pollutant in agricultural soils, with a site-level rate as high as 7.0% [7,8,9], and Cd soil pollution further shows an increasing trend from North to South China [10]. Field surveys showed that Cd concentrations in a considerable proportion of rice grains, especially in those grown in South China, exceeded the recommended food safety standard in the country [11,12,13]. One strategy to prevent Cd food contamination is to find and create more Cd low-accumulating cultivars of crops and vegetables using genetic breeding, and alleviation of Cd soil pollution can be achieved through phytoremediation utilizing high-accumulating plants. Therefore, understanding the physiological and molecular mechanisms of Cd uptake, transport, and accumulation by plants is of great significance for formulating strategies for phytoremediation of Cd-contaminated soils or prevention of Cd accumulation in crops.

An increasing number of studies have been conducted on the Cd migration pathway in plants, providing detailed information on the mechanism of Cd transport. There are four major processes that mediate Cd transport from roots to shoots: (1) root uptake; (2) loading into the root xylem; (3) long-distance translocation via the xylem and phloem pathways; (4) phloem re-translocation [14,15]. Plants absorb heavy metals by either active or passive absorption, with the root tips being the main Cd-absorbing area [16]. As a non-essential element, Cd^2+^ can enter the root through ion channels permeable to essential elements such as Ca^2+^ and K^2+^ [17,18]. It can also enter plant cells actively via uptake systems for essential elements such as Zn and Fe [19]. After root absorption, loading into the root xylem is one of the most critical steps for Cd transport [14]. Cd^2+^ or various Cd chelates can complete xylem loading through the symplast or the apoplast pathways [16]. The symplast pathway uses plasmodesmata to transport heavy metals between the cells, finally transporting them to the central column. The apoplast pathway transports water and heavy metals through the intercellular spaces or the cell wall continuum [14,20]. After Cd is loaded into the root xylem, it needs to be transported through the xylem and phloem for long-distance transport to the shoots. Phloem can serve as a major transport route for long-distance source-to-sink Cd transport via Cd–phytochelatin (PC) and Cd–glutathione complexes [21]. In addition, the phloem is primarily responsible for nutrient re-translocation, and in the *Sedum alfredii* Hance hyperaccumulating ecotype (HE), efficient phloem transport retransfers Cd from old to young organs [22].

Many transporter protein families are involved in the process of plant Cd uptake from the soil to be re-transported through the phloem. Clarifying the functions of these transporters regulating Cd and its chelates is important to understand the molecular mechanisms of plant responses to Cd. Thus far, the identified Cd transporters mainly include members of the natural resistance-associated macrophage protein (NRAMP), heavy metal-transporting ATPases (HMA), zinc and iron regulated transporter protein (ZIP), ATP-binding cassette (ABC), and yellow stripe-like (YSL) families.

## 2. Natural Resistance-Associated Macrophage Proteins

Nramps represent a class of metal transporters widely present in plants that are mainly involved in the absorption and transport of Fe^2+^, Mn^2+^, Cd^2+^, and other metal ions [23,24]. The involvement of *Nramp* genes in Cd transport was first reported in the model plant *Arabidopsis thaliana*. In recent years, research has been focused on food crops such as *Oryza sativa*, *Triticum polonicum* and *Fagopyrum esculentum*, and hyperaccumulator plants have also been explored. These proteins have also been identified in other plants.

**Figure 1:**
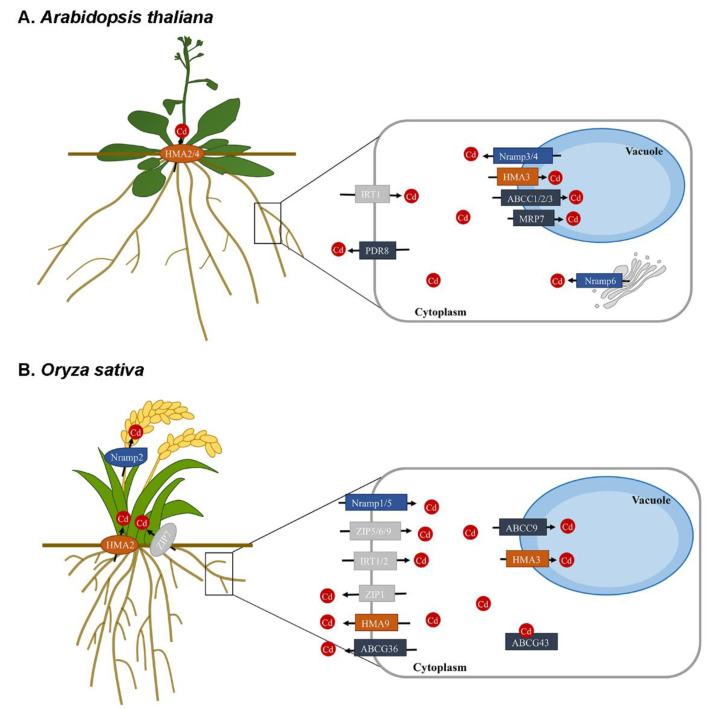
Uptake and transport of Cd. (**A**) In *Arabidopsis thaliana*, AtIRT1 is involved in Cd uptake by the roots. After Cd enters the root cells, it can be sequestered into the vacuole via AtHMA3, AtABCC1, AtABCC2, AtABCC3, and AtMRP7. AtNramp3 and AtNramp4 mediate the transport of Cd from the vacuole into the cytoplasm, while AtNramp6 transport Cd out of its storage compartment. AtHMA2 and AtHMA4 are involved in xylem loading to transport Cd to the shoots. Moreover, AtPDR8 mediates Cd efflux. (**B**) In *O. sativa*, OsNramp1, OsNramp5, OsZIP5, OsZIP6, OsZIP9, OsIRT1, and OsIRT2 are involved in Cd uptake by the rice roots. After Cd enters the root cells, it can be transported to the vacuoles, where it is sequestered, by OsHMA3 and OsABCC9. OsABCG43 also aids the sequestration of Cd in the roots. OsHMA2 and OsZIP7 are involved in xylem loading to transport Cd to the shoots. OsNramp2 mediates Cd re-translocation to the grains. Moreover, OsZIP1, OsHMA9, and OsABCG36 mediate Cd efflux in roots.

In *A. thaliana*, four *Nramp* genes have been found to be related to Cd transport. Overexpression of *AtNramp1* increased Cd sensitivity and accumulation in yeast (Table 1) [25]. *AtNramp3* and *AtNramp4* encode tonoplast-localized proteins, and yeast expressing the two genes showed an increased sensitivity to Cd (Figure 1, Table 1). Overexpression of *AtNramp3* in *Arabidopsis* conferred hypersensitivity to Cd [25,26,27,28], but overexpression of *AtNramp4* in *A. thaliana* only conferred a slight hypersensitivity to Cd [25,29]; *AtNramp3* and *AtNramp4* can also mediate the transport of Cd out of the vacuoles in *Arabidopsis* [25,28]. *AtNramp6* is a Cd transporter that can either transport Cd out of its storage compartment or into the toxic cellular compartment (Figure 1, Table 1) [30].

*Nramp* genes involved in the transport of Cd are mainly studied in rice among food crops. Three *Nramp* genes have been identified to be functionally associated with Cd. *OsNramp1,* a transporter localized in the plasma membrane responsible for Cd uptake and transport within plants, is mainly expressed in the roots and the leaves and is localized in all root cells except the central vasculature and in leaf mesophyll cells (Figure 1, Table 1) [32,33]. Tiwari et al. [31] observed that *OsNramp1* is involved in xylem-mediated loading and that it increased the accumulation of As and Cd in plants by heterologous expression of *OsNramp1* in *Arabidopsis*. However, Chang et al. [33] showed that *OsNramp1* transported Cd and Mn when expressed in yeast but did not transport Fe or As. Overexpression of *OsNramp1* in rice reduced Cd accumulation in the roots, but increased it in the leaves. Knockout of *OsNramp1* resulted in decreased Cd and Mn uptake by the roots and their accumulation in the shoots and the grains [32,33].

*OsNramp2* is localized in the tonoplast and mainly expressed in the embryo of germinating seeds, roots, leaf sheaths, and leaf blades (Figure 1, Table 1) [35]. The knockout of *OsNramp2* significantly decreased Cd concentration in the grains, but increased it in the leaves and the straws, suggesting that it mediates Cd efflux from the vacuoles in the vegetative tissues for translocation to the grains [34,35].

*OsNramp5* encodes a plasma membrane protein polarly localized at the distal side of both exodermis and endodermis cells, and responsible for the influx of Mn and Cd into root cells from external solutions (Figure 1, Table 1) [37,38]. Knockout of *OsNramp5* significantly reduced Cd concentration in the roots and shoots [38,39]. In a Cd-contaminated paddy field experiment, it was found that Cd concentration in the grains of the knockout line was much lower than that of the wild-type (WT) [39]. Surprisingly, the overexpression of *OsNramp5* enhanced Cd root uptake, but significantly reduced its accumulation in the shoots and grains. Xylem loading was also disturbed in *OsNramp5*-overexpressing plants, with a reduced translocation from the roots to the shoots [36].

In *Triticum polonicum* L and *Triticum turgidum* L, *TpNramp3, TpNramp5,* and *TtNramp6* encode plasma membrane proteins (Table 1). Overexpression of *TtNramp6* increased Cd concentration and its accumulation in the whole plant of *Arabidopsis* [42]. Overexpression of *TpNramp3* or *TpNramp5* also increased the concentrations of Cd, Co, and Mn in the whole plant [40,41]. In *Hordeum vulgare*, *HvNramp5* encodes a plasma membrane-localized transporter required for the uptake of Cd and Mn, but not Fe (Table 1), that presents 84% identity with *OsNramp5*. *HvNramp5* was mainly expressed in the roots, with higher expression levels in the root tips than in the basal region [43]. Knockout of *HvNramp5* in barley resulted in reduced concentrations of Mn and Cd in the roots and shoots but did not change the concentrations of other metals [43]. In *Fagopyrum esculentum* Moench, the plasma membrane-localized transporter *FeNramp5* is responsible for the uptake of Mn and Cd (Table 1). *FeNramp5* can also complement the phenotype of an *AtNramp1 Arabidopsis* mutant in terms of growth and accumulation of Mn and Cd [44]. *BnNramp1b* is localized in the plasma membrane and can transport Cd (Table 1) [45]. Yue et al. demonstrated that *BcNramp1* plays a role in Cd influx of *Arabidopsis* root cells using noninvasive microelectrode ion flux measurements (Table 1) [46].

Studies on *Nramp* Cd-transporting genes in hyperaccumulator plants are mainly focused on *Noccaea caerulescens* (*Thlaspi caerulescens*) and *Sedum alfredii* Hance. In *N. caerulescens*, *NcNramp1* participates in the influx of Cd across the endodermal plasma membrane and thus may play an important role in the Cd flux into the stele and its root-to-shoot translocation (Table 1) [47]. *TcNramp3* and *TcNramp4* are localized in the tonoplast (Table 1). *TcNramp3* or *TcNramp4* expression rescued Cd and Zn hypersensitivity induced by the inactivation of *AtNramp3* and *AtNramp4* in *Arabidopsis* [48]. Additionally, in overexpression tobacco lines, the roots were found to be more sensitive to Cd [49]. In the *S. alfredii* Hance, the plasma membrane-localized *SaNramp1* transporter is highly expressed in the young tissues of the shoots (Table 1), and its overexpression in tobacco significantly increased Cd concentration at this location [50]. Ectopic expression of *SaNramp3* in *Brassica juncea* enhanced Cd root-to-shoot translocation (Table 1), thus increasing Cd accumulation in the shoots [51]. Overexpression of *SaNramp6*, localized in the plasma membrane, increased Cd uptake and accumulation in *A. thaliana* (Table 1) [52]. Employing site-directed mutagenesis and functional analysis of mutants in yeast and *Arabidopsis*, the conserved L157 site in *SaNramp6h* was found to be critical for metal transport [53].

*Nramp* genes have also been identified in other plants. *MxNramp1* (localized in the plasma membrane) and *MxNramp3* (localized in the tonoplast) can transport Cd in yeast (Table 1) [54]. In *Malus hupehensis*, overexpression of *MhNramp1* increases Cd uptake and accumulation, thereby exacerbating cell death (Table 1) [55]. *SpNramp1, SpNramp2*, and *SpNramp3* are plasma membrane-localized transporters in *Spirodela polyrhiza* (Table 1), and overexpression of *SpNramp1* or *SpNramp2* increased Cd accumulation [56]. Similarly, overexpression of *CjNramp1* in *Arabidopsis* resulted in high tolerance to Cd (Table 1) [57]. Furthermore, overexpression of *NtNramp1* in tobacco could promote Cd uptake and Fe transportation (Table 1) [58], and the tonoplast-localized *NtNramp3* transporter was found to be involved in the regulation of Cd transport from the vacuole to the cytoplasm using CRISPR/Cas9 technology (Table 1) [59].

## 3. Heavy Metal Transporting ATPases

HMAs play an important role in absorbing and transporting essential metal ions, such as Cu^2+^, Co^2+^ and Zn^2+^, by ATP hydrolysis; they can also transport Cd^2+^ and Pb^2+^. HMAs can be divided into two classes: those transporting monovalent cations (Cu, Ag) and those transporting divalent cations (Zn, Co, Cd, Pb) [60]. First described in *A. thaliana*, they have been studied more in food crops and hyperaccumulator plants in recent years due to their strong capacity to transport Cd; they have also been slightly less researched in other plants.

**Table 2:** Genes encoding Heavy Metal transporting ATPases (HMAs) for Cd transport in plants.

Plant Species	Genes	Expression Sites	Subcellular Location	Function	References
*Arabidopsis thaliana*	*AtHMA2*	-	Plasma membrane	Cd translocation	[61,62]
	*AtHMA3*	Root apex	Tonoplast	Cd sequestration	[63,64]
	*AtHMA4*	tissues surrounding the root vascular vessels	Plasma membrane	Cd translocation	[61,65,66,67]
*Oryza sativa* L	*OsHMA2*	in the mature zone of the roots at the vegetative stage	Plasma membrane	Cd translocation	[68,69,70]
	*OsHMA3*	Roots	Tonoplast	Cd sequestration	[71,72,73,74,75,76]
	*OsHMA9*	vascular bundles and anthers	Plasma membrane	Cd efflux	[77]
*Triticum aestivum* L.	*TaHMA2*	Nodes	Plasma membrane	Cd translocation	[78]
*Glycine max*	*GmHAM3w*	Roots	Endoplasmic reticulum (ER)	Cd sequestration	[79]
*Sedum plumbizincicola*	*SpHMA1*	Leaves	Chloroplast envelope	Cd efflux	[80]
	*SpHMA3*	Leaves	Tonoplast	Cd sequestration	[81]
*Sedum alfredii* Hance	*SaHMA3*	Shoots	Tonoplast	Cd sequestration	[82]
*Thlaspi caerulescens*	*TcHMA3*	Roots and shoots	Tonoplast	Cd sequestration	[83]
	*TcHMA4*	Roots		-	[84]
*Brassica juncea*	*BjHMA4*	Roots, stems and leaves	Cytosol	Cd translocation	[85]
*Iris lactea*	*IlHMA2*	Roots	Plasma membrane	Cd translocation	[86]
*Populus tomentosa* Carr.	*PtoHMA5*	-	-	Cd translocation	[87]

*AtHMA2*, *AtHMA3*, and *AtHMA4* are reportedly associated with Cd transport in *A. thaliana*. *AtHMA3* encodes a tonoplast-localized transporter that plays a role in Cd, Zn, Co, and Pb detoxification (Figure 1, Table 2) [64]. Overexpression of *AtHMA3* enhanced Cd tolerance and increased its accumulation [63,64]. *AtHMA2* and *AtHMA4*, localized in the plasma membrane, are responsible for the xylem loading of Zn/Cd and play a key role in their accumulation in the shoots (Figure 1, Table 2) [61,62,65]. Ceasar et al. [66] found that the di-cysteine residues at the C-terminus of *HMA4* in *A. thaliana* were only partially required for Cd transport. Furthermore, ectopic expression of 35S::*AtHMA4* reduced Cd accumulation due to the induction of the apoplastic barrier in tobacco [67].

The study of the HMA family is predominantly focused on food crops. Three Cd-transport associated *HMA* genes were identified in the genome of rice, one of the major food crops. The plasma membrane-localized transporter *OsHMA2* is involved in the root-to-shoot translocation of Zn and Cd (Figure 1, Table 2). *OsHMA2* is mainly expressed in the mature zone of the roots at the vegetative stage, with the C-terminal region being essential for Zn/Cd translocation into the shoots [68,69]. Moreover, at the reproductive stage, *OsHMA2* also showed a high expression in the nodes. Knockout of *OsHMA2* resulted in reduced Zn and Cd concentrations in the upper nodes and reproductive organs compared with the WT, suggesting that *OsHMA2* participates in the transport of Zn and Cd through the phloem to developing tissues [70]. *OsHMA3* is localized in the tonoplast and sequestrates Cd into the root vacuoles to reduce its translocation, thereby mitigating Cd poisoning (Figure 1, Table 2) [71,72,73,74]. Silencing of *OsHMA3* resulted in increased root-to-shoot Cd translocation, whereas *OsHMA3* overexpression markedly decreased root-to-shoot Cd translocation and increased Cd tolerance, while greatly reducing its concentration in the grains [72,75]. The C-terminal region, and particularly the region containing the first 105 amino-acids, has an important role in the activity of *OsHMA3* [76]. *OsHMA9* encodes a heavy metal (Cd, Cu, Zn, and Pb) efflux protein present in the plasma membrane (Figure 1, Table 2). Knockout of *OsHMA9* results in higher Cd accumulation in the shoots compared with that of the WT, thus making the mutant sensitive to Cd [77]. Moreover, in *Triticum aestivum* L., overexpression of *TaHMA2* improved the root-shoot Zn/Cd translocation (Table 2) [78]. In *Glycine max* (soybean), *GmHAM3w* restricts Cd to the endoplasmic reticulum, where it is localized, and in the roots to limit translocation to the shoots (Table 2). Overexpression of *GmHMA3w* increased Cd concentration in the roots and decreased it in the shoots [79].

As a popular tool for the remediation of Cd-contaminated soils, there have been many studies on *HMA* genes with Cd transport and sequestration functions in hyperaccumulator plants in recent years. *SpHMA1* is an important efflux transporter localized in the chloroplast envelope and is responsible for exporting Cd from the chloroplast (Table 2), thus preventing Cd accumulation in *Sedum plumbizincicola*. Significantly increased Cd concentration in chloroplasts in *SpHMA1* RNAi transgenic plants and CRISPR/Cas9-induced mutants compared to WT [80]. *SpHMA3*, localized in the tonoplast and expressed mainly in the shoots (Table 2), plays an important role in Cd detoxification in young leaves by sequestering Cd into the vacuole [81]. In *S. alfredii*, the tonoplast-localized transporter *SaHMA3* is mainly expressed in shoots (Table 2). Its overexpression in tobacco significantly enhanced Cd tolerance and accumulation and greatly increased Cd sequestration in the roots [82]. Increased amounts of Cd were sequestered in the roots, but not in the leaf vacuoles, probably due to the heterologous expression. *TcHMA3* is a tonoplast-localized transporter responsible for Cd sequestration into the leaf vacuoles in *Thlaspi caeulescens* (Table 2) [83]. *TcHMA4* is involved in the active efflux of a large number of different heavy metals (Cd, Zn, Pb, and Cu) out of the cell (Table 2), with the C-terminus of the TcHMA4 protein being essential for heavy metal binding [84]. Moreover, *BjHMA4R* can significantly improve Cd tolerance and accumulation at low heavy metal concentrations by specifically binding to Cd^2+^ in the cytosol (Table 2) [85]. In other plants, *IlHMA2* is a plasma membrane transporter involved in Cd root-to-shoot translocation (Table 2). The genes regulating Zn homeostasis were significantly down regulated in *IlHMA2*-silenced lines, compared with that in WT [86]. *PtoHMA5* also participates in Cd root-to-shoot translocation (Table 2) [87].

## 4. ATP-Binding Cassette

This protein superfamily is one of the largest known superfamilies, with over 120 members in both *A. thaliana* and *O. sativa*. ABC transporters comprise four core domains (two nucleotide-binding and two transmembrane domains) [88] and are located in the plasma, vacuolar, and mitochondrial membranes, where they facilitate the transmembrane transport of substances via active transport [89,90,91,92]. The ABC family is further divided into 13 subfamilies, according to the size and domains of their members; the subfamilies involved in the transport of Cd and its chelates include the multidrug resistance-associated protein (MRP), pleiotropic drug resistance (PDR), and ABC transporter of the mitochondrion (ATM) subfamilies [93]. The current research on these three subfamilies is mainly focused on *A. thaliana* and *O. sativa*.

In *A. thaliana*, *AtABCC1* and *AtABCC2*—two important tonoplast transporters—play an essential role in sequestering the PC–Cd(II) complexes to the vacuoles (Figure 1, Table 3), thereby reducing the metal concentration in the root cells and its translocation to the shoots [92]. *AtABCC3* is involved in the vacuolar transport of the PC–Cd complexes (Figure 1, Table 3), with its activity being regulated by Cd and coordinated with the function of *AtABCC1/AtABCC2* [94]. The expression levels of *AtMRP6/AtABCC6* are significantly upregulated under Cd stress (Table 3) [95]. Overexpression of *AtMRP7,* which is localized in both the tonoplast and the plasma membrane (Figure 1, Table 3), increased Cd concentration in the leaf vacuoles and its retention in the roots in tobacco [96]. *AtPDR8*, located in the plasma membrane and the root epidermal cells, is an important efflux transporter that increases Cd tolerance by effluxing Cd^2+^ out of the root epidermal cells (Figure 1, Table 3). Overexpression of *AtPDR8* improved Cd tolerance but did not affect its accumulation or that of Pb [91]. *AtATM3* is a transporter localized in the mitochondrial membrane (Table 3), and its overexpression improved Cd tolerance and accumulation by increasing the biogenesis of Fe-S clusters and exporting them from the mitochondria into the cytosol in *Arabidopsis* [90]. Overexpression of *AtATM3* in *B. juncea* conferred enhanced Cd and Pb tolerance by inducing the expression of its glutathione synthetase II (BjGSHII) and phytochelatin synthase 1 (BjPCS1) enzymes [97].

In *O. sativa*, *OsABCC9* was predominantly expressed in the root stele after Cd treatment (Figure 1, Table 3). It mainly mediates Cd accumulation by sequestering of Cd into the root vacuoles, thereby reducing its translocation to the shoots and grains [98]. The plasma membrane-localized *OsABCG36* transporter functions as a Cd extrusion pump (Figure 1, Table 3), thus increasing Cd tolerance by exporting it or its conjugates from the root cells in rice. Compared with the WT, *OsABCG36* knockout had a significantly higher Cd accumulation in the root cell sap and significantly increased sensitivity to Cd [99]. Yeast heterologous expression indicated that *OsABCG43* and *OsABCG48* conferred Cd tolerance (Figure 1, Table 3); overexpression of *OsABCG48* in rice reduced Cd concentration in the roots [100,101]. Similarly, in *Triticum aestivum*, *TaABCC13* was reportedly involved in Cd uptake and transport (Table 3), as Cd concentration in the roots and shoots of *TaABCC13*:RNAi line decreased, compared with that of the WT [102].

In other plants, some ABC genes have also been found to have a Cd-transporting role. Yeast-expressed *RgABCC1*, found in *Rehmannia glutinosa*, increased Cd tolerance (Table 3) [103]. Similarly, *PtoABCG36* reduced Cd concentration in plants by mediating its efflux (Table 3), thereby improving Cd tolerance [104].

## 5. Zinc- and Iron-Regulated Transporter Proteins

There are many members in the ZIP family, with all of them generally presenting eight transmembrane regions and metal ion-binding conserved domains that play a role in their transport. Not only can they transport essential metal ions such as Fe^2+^ and Zn^2+^, but also Cd^2+^ [105]. The first member of the ZIP family to be described was *NcZNT1,* found in *N. caerulescens* (Table 4) [106]. Overexpression of *NcZNT1* enhanced the tolerance and accumulation of Zn and Cd in *Arabidopsis*, suggesting its involvement in the long-distance translocation of xylem loading from the roots to the shoots [107].

In recent years, studies on the role of the ZIP family in Cd transport have mainly focused on *O. sativa*. *OsIRT1* and *OsIRT2* are the major transporters participating in Fe and Cd uptake as observed in an heterologous expression experiment in yeast (Figure 1, Table 4) [110]. The IRT1 protein, first described in *A. thaliana*, mediates the absorption of a variety of metals including Fe, Zn, and Cd (Figure 1, Table 4) [108,109]. Similarly, *IRT1* has also been explored in pea seedlings, mulberry (*Morus* L.), *Triticum polonicum* L., and *Hordeum vulgare*. Overexpression of *IRTI* in *Arabidopsis* and rice increased their sensitivity to Zn and Cd [110,118,123,124,125,126]. *OsZIP1*, a metal efflux transporter, is localized in the endoplasmic reticulum and the plasma membrane and is mainly expressed in the roots (Figure 1, Table 4). Overexpression of *OsZIP1* protects rice plants from an excess of Zn, Cu, and Cd by limiting metal accumulation in their tissues [111]. Plasma membrane-localized proteins OsZIP5 and OsZIP9 have influx transporter activity that functions synergistically in the Zn/Cd uptake in rice (Figure 1, Table 4). Overexpression of *OsZIP9* markedly increased the Zn/Cd levels in the aboveground tissues in brown rice. *OsZIP9* is also responsible for the uptake of Zn and Co into the root cells [112,115]. Employing electrophysiological techniques, Kavitha et al. [113] demonstrated the uptake of Cd by *OsZIP6* (Figure 1, Table 4). *OsZIP7* encodes a plasma membrane-localized protein responsible for Cd/Zn influx and is expressed in the parenchyma cells of vascular bundles in the roots and nodes (Figure 1, Table 4). Compared with the WT, an *OsZIP7* knockout results in Zn and Cd retention in the roots and the basal ganglia, hindering their upward transmission and thus playing a role in xylem loading in the roots and inter-vascular transfer in the nodes to deliver Zn/Cd to the grains in rice [114].

ZIP genes related to Cd transport have also been reported in other plants. In *Nicotiana tabacum*, *NtZIP4A* and *NtZIP4B* are two copies of *ZIP4*, with 97.57% homology at the amino acid level. *NtZIP4A/B* is a plasma membrane-localized transporter that regulates Zn and Cd translocation from the roots to the shoots (Table 4) [116,117]. Similarly, *MaZIP4* is also localized in the plasma membrane and has Cd transport activity (Table 4) [118].

## 6. Yellow Stripe-Like Proteins

The YSL family mediates the transmembrane transport of metal ions and chelates formed by metal ions and nicotinamide in plants, as well as the long-distance transport from the roots to the shoots [105]. YSL proteins were first reported to have a role in Fe transport, and then were subsequently found to participate in the transport of Cu, Zn, Cd, and Mn [127]. Members of this family involved in Cd transport include *YSL1, YSL3*, *YSL6*, and *YSL7*. *MsYSL1* and *SnYSL3* are plasma membrane-localized transporters responsible for long-distance Cd translocation from the roots to the shoots (Table 4). An excess of Cd reportedly stimulated their expression. Overexpression of *MsYSL1* or *SnYSL3* in *Arabidopsis* increased the Cd translocation ratio under Cd stress [119,120]. *VcYSL6* is located in the chloroplast, and its expression is up-regulated under Cd induction (Table 4). Overexpression of *VcYSL6* in tobacco increased Cd concentrations in the leaves [121]. *BjYSL7* encodes a plasma membrane-localized metal–nicotinamide transporter (Table 4). The concentrations of Cd and Ni in the shoots of *BjYSL7*-overexpressing transgenic tobacco plants are significantly higher than that of WT plants, suggesting a role of *BjYSL7* in Cd translocation from the roots to the shoots [122].

## 7. Conclusions and Further Perspectives

In this review, we outlined the role of transporters in the uptake and transport of Cd by plants. After long-term evolution, plants have formed a set of complex mechanisms to cope with Cd stress. The key role of transporters in it has also been confirmed by multiple studies, and excellent progress has been made in determining the localization, specific expression, and function of each protein family member. However, the regulatory network for Cd uptake and transport in plants is extremely large and involves multiple genes. For example, in *O. sativa*, OsZIP5 and OsZIP9 are tandem duplicates and act synergistically in Cd uptake [112]. OsNRAMP1 and OsNRAMP5 are involved in Cd uptake via roots and knocking out both these genes resulted in large decreases in the uptake of Cd, compared to the case for the knockout of either one of genes [33]. However, the functions of many genes and the relationships between them are still unknown. Therefore, the traditional way of examining a single gene can no longer meet the requirements of the post-genomic era, and the mutual synergy between functional genes should be explored further in future research. Moreover, unknown genes related to plant Cd uptake and transport and the synergistic relationship between these genes can be further explored by constructing mutants and using molecular biology techniques in future studies. This would contribute to our understanding of the vast regulatory network of genes involved in Cd uptake, translocation and accumulation. In addition, studying the functions of various genes and the mechanisms underlying these functions would help cultivate Cd-tolerant plants using transgenic technology, which would further be helpful to restore Cd-contaminated soil.

## Figures and Tables

**Table 1:** Genes encoding Natural Resistance-Associated Macrophage Proteins (Nramp) for Cd transport in plants.

Plant Species	Genes	Expression Sites	Subcellular Location	Function	References
*Arabidopsis thaliana*	*AtNramp1*	Roots	Plasma membrane	-	[25]
	*AtNramp3*	Roots and aerial parts	Tonoplast	Cd transport	[25,26,27,28]
	*AtNramp4*	Roots and aerial parts	Tonoplast	Cd transport	[25,28,29]
	*AtNramp6*	Seed embryo, lateral roots and young leaves	Golgi/trans-Golgi network	Cd transport	[30]
*Oryza sativa* L.	*OsNramp1*	Roots and leaves	Plasma membrane	Cd uptake and translocation	[31,32,33]
	*OsNramp2*	Embryo of germinating seeds, roots, leaf sheaths and leaf blades	Tonoplast	Cd retranslocation	[34,35]
	*OsNramp5*	Roots epidermis, exodermis, outer layers of cortex and tissues around xylem	Plasma membrane	Cd uptake	[36,37,38,39]
*Triticum polonicum* L.	*TpNramp3*	leaf blades and roots at the jointing and booting stages, first nodes at the grain filling stage	Plasma membrane	Cd accumulation	[40]
	*TpNramp5*	Roots and basal stems of DPW	Plasma membrane	Cd accumulation	[41]
*Triticum turgidum* L.	*TtNramp6*	Roots	Plasma membrane	Cd accumulation	[42]
*Hordeum vulgare*	*HvNramp5*	Roots	Plasma membrane	Cd uptake	[43]
*Fagopyrum esculentum* Moench	*FeNramp5*	Roots	Plasma membrane	Cd uptake	[44]
*Brassica napus*	*BnNramp1b*	Vegetative tissue, flowers and siliques	-	-	[45]
*Brassica rapa* L.	*BcNramp1*	Roots	Plasma membrane	Cd uptake	[46]
*Noccaea caerulescens* (*Thlaspi caeulescens*)	*NcNramp1*	Roots and shoots	Plasma membrane	-	[47]
	*TcNramp3*	-	Tonoplast	-	[48,49]
	*TcNramp4*	-	Tonoplast	-	[48]
*Sedum alfredii* Hance	*SaNramp1*	Young tissues of the shoots	Plasma membrane	Cd translocation	[50]
	*SaNramp3*	-	-	Cd translocation	[51]
	*SaNramp6*	Roots	Plasma membrane	Cd uptake or translocation	[52,53]
*Malus xiaojinensis*	*MxNramp1*	Roots	Plasma membrane	Cd uptake and translocation	[54]
	*MxNramp3*	Roots and leaves	Tonoplast	Cd uptake and translocation	[54]
*Malus hupehensis*	*MhNramp1*	Roots	Cell membrane	Cd uptake	[55]
*Spirodela polyrhiza*	*SpNramp1*	-	Plasma membrane	Cd accumulation	[56]
	*SpNramp2*	-	Plasma membrane	Cd accumulation	[56]
	*SpNramp3*	-	Plasma membrane	-	[56]
*Crotalaria juncea*	*CjNramp1*	Leaves, stems, and roots	Plasma membrane	Cd uptake and translocation	[57]
*Nicotiana tabacum*	*NtNRAMP1*	Roots	-	Cd uptake	[58]
	*NtNRAMP3*	Conductive tissue of leaves	Tonoplast	Cd efflux	[59]

**Table 3:** Genes encoding ATP-Binding Cassette (ABC) for Cd transport in plants.

Plant Species	Genes	Expression Sites	Subcellular Location	Function	References
*Arabidopsis thaliana*	*AtABCC1*	-	Tonoplast	Cd sequestration	[92]
	*AtABCC2*	-	Tonoplast	Cd sequestration	[92]
	*AtABCC3*	-	-	Cd sequestration	[94]
	*AtMRP6/AtABCC6*	Xylem-opposite pericycle cells where lateral roots initiate		-	[95]
	*AtMRP7*	-	Plasma membrane and tonoplast	Cd sequestration	[96]
	*AtPDR8*	Root epidermal cells	Plasma membrane	Cd efflux	[91]
	*AtATM3*	Roots	Mitochondrial membrane	-	[90,97]
*Oryza sativa* L.	*OsABCC9*	Root stele	Tonoplast	Cd sequestration	[98]
	*OsABCG36*	Roots	Plasma membrane	Cd efflux	[99]
	*OsABCG43*	Roots	-	Cd sequestration	[100]
	*OsABCG48*	-	-	-	[101]
*Triticum aestivum*	*TaABCC13*	-	-	Cd uptake and transport	[102]
*Rehmannia glutinosa*	*RgABCC1*	Roots	-	-	[103]
*Populus tomentosa*	*PtoABCG36*	Roots	Plasma membrane	Cd efflux	[104]

**Table 4:** Genes encoding Zinc and Iron regulated transporter Protein (ZIP) and Yellow Stripe-Like proteins (YSL) for Cd transport in plants.

Plant Species	Genes	Expression Sites	Subcellular Location	Function	References
**Genes encoding Zinc and Iron regulated transporter Protein (ZIP)**					
*Noccaea caerulescens* L.	*NcZNT1*	roots and shoots	-	-	[106,107]
*Arabidopsis thaliana*	*AtIRT1*	Roots	Plasma membrane	Cd uptake	[108,109]
*Oryza sativa* L.	*OsIRT1*	Roots	Plasma membrane	Cd uptake	[108,109,110]
	*OsIRT2*	Roots	Plasma membrane	Cd uptake	[110]
	*OsZIP1*	Roots	Endoplasmic reticulum (ER) and plasma membrane	Cd efflux	[111]
	*OsZIP5*	Roots	Plasma membrane	Cd uptake	[112]
	*OsZIP6*	Shoots and roots	-	Cd uptake	[113]
	*OsZIP7*	parenchyma cells of vascular bundles in roots and nodes	Plasma membrane	Cd translocation	[114]
	*OsZIP9*	Roots	Plasma membrane	Cd uptake	[112,115]
*Nicotiana tabacum* var Xanthi	*NtZIP4A/B*	Leaves and roots	Plasma membrane	Cd translocation	[116,117]
*Morus alba*	*MaZIP4*	-	Plasma membrane	-	[118]
**Genes encoding Yellow Stripe-Like proteins (YSL)**					
*Miscanthus sacchariflorus*	*MsYSL1*	Stems	Plasma membrane	Cd translocation	[119]
*Solanum nigrum*	*SnYSL3*	Vascular tissues and epidermal cells of the roots and stems	Plasma membrane	Cd translocation	[120]
*Vaccinium* ssp.	*VcYSL6*	-	Chloroplast	-	[121]
*Brassica juncea*	*BjYSL7*	Stems	Plasma membrane	Cd translocation	[122]

## Data Availability

Not applicable.
